# Measurements of Spontaneous Communication Initiations in Children with Autism in Preschool through Third Grade Classrooms

**DOI:** 10.1007/s10803-022-05738-1

**Published:** 2022-10-12

**Authors:** Sandy Luong Birkeneder, Nicole Sparapani

**Affiliations:** School of Education and the UC Davis MIND Institute, University of California, Davis, One Shields Ave, 95616 Davis, CA USA

**Keywords:** Autism, Spontaneous communication, Classrooms, Communicative functions, Communication initiations, Rate of communication

## Abstract

We utilized classroom video observations to examine the frequency and function of spontaneous communication in 112 preschool–3rd grade children with autism within 57 classrooms. Children initiated 7.53 instances (*SD* = 9.42) of spontaneous communication on average within a 12-minute sample, a rate of 0.69 initiations per minute. Autism features, receptive and expressive language, and adaptive functioning were associated with communication rate. A 4-factor model of spontaneous communication functions exhibited the best relative and absolute fit to the data. Findings highlight, and begin to explain, variability in spontaneous communication children used in classrooms, link individual developmental characteristics to communicative initiations, and provide evidence for conceptualizing and measuring spontaneous communication in learners with autism across classroom activities. Implications and future directions are discussed.

Studies have described spontaneous communication initiations (spontaneous communication) as communication directed toward another person, unprompted (Forde et al., 2011; Rama et al., 2014; Stone & Caro-Martinez 1990; Wetherby et al., 1998). Across studies, measuring the frequency of spontaneous communication in children with autism spectrum disorder (autism) has helped to characterize their diagnostic features, provided evidence of intervention effectiveness (Koegel et al., 2003; Whalen et al., 2006), and contributed to assessments of social communication development (Duffy & Healy, 2011; Srinivasan et al., 2016; Sutton et al., 2019). However, studies evaluating spontaneous communication in children with autism have often been carried out in controlled clinical settings during early childhood years (e.g., Drew et al., 2007; Wetherby & Prizant, 2002). Less frequent are studies examining communication within naturalistic, classroom settings. Hence, there is not currently a common metric for evaluating spontaneous communication within classrooms for children with autism. Studies, therefore, have resorted to alternative or novel methods for measuring spontaneous communication (e.g., Clifford et al., 2010; Kim et al., 2014; Grzadzinski et al., 2016), making replication and generalization of findings across settings and populations difficult.

Children with autism exhibit heterogeneous developmental and skill profiles that can lead to differences in the type and frequency of spontaneous communication across the population. Adding an extra layer of complexity, classrooms are difficult settings to measure spontaneous communication due to the number of variables that might influence the frequency and function of initiations, such as the instructional context, communicative partner, or classroom activity. Although, many of these factors are better controlled for within clinical settings, there is merit in examining spontaneous communication within classrooms. Studies have argued that moving interventions from clinical to classroom settings, for example, may have long-term outcomes and resource sustainability benefits (e.g., Sutton et al., 2019). Others have suggested that clinical environments might be set up to promote or facilitate communication, which might lead to an increase in child initiations (Hauck et al., 1995). Whether these higher frequencies can translate into actual classrooms is an area that needs further attention.

Understanding how and why school-age children with autism spontaneously communicate in classrooms may provide a means to understand and monitor child talk within classroom activities. This knowledge might also provide insight into missed learning opportunities and potential instructional barriers that limit children’s communication, such as specific types of activities or instructional contexts. This is particularly relevant since the majority of children with autism are educated within the public school system and spend a large proportion of their day within classrooms (Department of Education, 2015). Hence, the purpose of this study was to examine the amount and type of spontaneous communication that children with autism initiate within academic and nonacademic classroom activities, explore the associations among child characteristics (receptive and expressive language, adaptive behavior, and autism features) with rate of spontaneous communication, and evaluate the measurement model of spontaneous communication functions. Evaluating autistic children’s intentional communication within real-world contexts is an area of research that has been given little attention overall. It is our hope that this study will provide valuable information to researchers that lays the groundwork for future study (i.e., describing communication and providing a common metric) while simultaneously suggesting potential recommendations that practitioners might consider when programing for children with autism in their classrooms.

## Measuring and Conceptualizing Spontaneous Communication

**Measuring Spontaneous Communication**. Standardized tools, such as the *Early Social Communication Scales* (ESCS; Mundy et al., 2003) and *the Communication and Symbolic Behavior Scales* (CSBS; Wetherby & Prizant 2002) are two measures among others that have been used to assess spontaneous communication in children with autism more broadly. The *Autism Diagnostic Observation Schedule*–Generic (ADOS-G, Lord et al., 2000) also provides a measure of spontaneous communication within the Social Affect domain. Tools such as these, however, are commonly performed in clinical or controlled settings, which might not provide an accurate assessment of a child’s abilities within real classrooms (Clifford et al., 2010). Standardized questionnaires are also often used to assess social and communication behaviors (Chen et al., 2018). Questionnaires such as the Vineland Adaptive Behavior Scales, Second Edition (VABS-II; Sparrow et al., 2005) and the Social Responsiveness Scale (SRS-2; Constantino & Gruber 2012), for example, provide a broad measure of communication and can be used by non-clinicians such as parents, teachers, and caregivers, which strengthens overall utility of the assessment. However, the information gleaned from such tools is limited to reporting child communication rather than direct observation or individual assessment of child communication.

In addition to the standardized measures and questionnaires listed above, most studies examining spontaneous communication have developed observational coding systems in their analysis of the construct. For example, a recent study examined the psychometric properties of the Autism Peer Interaction Observation Scale, which assesses spontaneous interactions that include social communication behaviors in preschoolers (Bauminger-Zviely & Shefer, 2021). Yet the APIO and other similar studies assessing spontaneous communication vary by instructional context, type of activity, and/or children’s age range (Howlin et al., 2007; Forde et al., 2011; Kossyvaki & Guldberg, 2012; Pasco et al., 2008). Few studies have focused on spontaneous communication in naturalistic environments (i.e., Stone & Caro-Martinez 1990). Those that have were carried out within early childhood or special education classrooms during unstructured, nonacademic activities, such as play and mealtime (Dykstra & Watson, 2015; Hauck et al., 1995; Kim et al., 2014). Few examined children’s spontaneous communication during structured, academic activities, across classroom settings, with different interacting partners.

**Conceptualizing Spontaneous Communication**. Conceptualizing spontaneous communication in children with autism has also varied across studies. Although there is consistency in how spontaneous communication has been operationalized—acts of unprompted and intentional communication directed toward another person to serve a function—the type of act (e.g., verbal, nonverbal), who it was directed toward (e.g., adult, peer), and the function it serves (e.g., requesting, protesting, etc.) have varied across studies. For example, in an early study Watson and colleagues (1989) examined spontaneous communication within preschool children as nine varying communicative functions, including getting attention, social routines, requesting, commenting, rejecting/refusing, giving information, seeking information, expressing feelings, and social interactions. The authors also measured to who children directed the acts toward and what form of communication they used (motoric acts, speech, vocalizations, gestures). In contrast, Sparapani and colleagues (2016) examined spontaneous communication in a sample of kindergarten–2nd grade children with autism, measuring spontaneous communication as a frequency count of all directed initiations that served a function.

Studies evaluating young children with autism in clinical and classroom settings that have specified communicative functions have frequently conceptualized the construct according to three broad functions; behavior regulation, social communication, and joint attention (e.g., Dykstra & Watson, 2015; Maljaars et al., 2011; Shumway & Wetherby, 2009). In an early study, Wetherby and colleagues (1989) described these functions as three developmental levels of spontaneous communication initiations within a sample of young children with autism. Studies have drawn from and expanded upon these three developmental levels to examine spontaneous communication in children with autism across educational contexts. Clifford and colleagues (2010), for example, examined 4- to 6-year-olds within general and special education classrooms and extended their conceptualization of spontaneous communication to include nonverbal showing and giving behaviors as well as varying types of verbal communication (vocalizations, single words, and phrases). It may be, however, that describing spontaneous communication within these three developmental levels is too narrow for older, elementary-aged children with autism who may communicate for a broader range of functions.

## Associations with Child Characteristics

Understanding how and why children with autism communicate within classrooms might also allow for better targeting of communication development. Several studies have examined the relationship between child communication and later development (e.g., Brooks & Meltzoff 2005; 2008) as well as the link between child characteristics in relation to communication development overall or in comparison to other groups (e.g., Baldwin & Moses 2001; Drew et al., 2007; Prizant & Laurent, 2003). However, few studies of recent have examined how child characteristics relate to spontaneous communication (Loveland & Landry, 1986; Stone & Caro-Martinez, 1990). There is still much to learn about specific child characteristics that are associated with spontaneous communication—an area that this paper specifically addresses.

### Study Purpose and Research Objectives

In this study, we utilized archival classroom video observations, recorded at the beginning of the school year, to examine the frequency, function, and direction of spontaneous communication within a large sample of preschool–third grade children with autism as they engaged with their teachers and peers during various academic (literacy, math, other academics) and nonacademic (arts & crafts, snack, recreation) classroom activities. We explored the associations among child characteristics including, receptive and expressive language, adaptive behavior, and autism features, with rate of spontaneous communication. Finally, we used confirmatory factor analysis (CFA) to examine the factor structure of spontaneous communication functions within classroom activities as guided by the extant literature.

## Methods

### Participants

Participants were recruited for a longitudinal study evaluating the efficacy of a school-based intervention for children with autism. Approval was received by the University Institutional Review Board prior to the start of the longitudinal study. Video-recorded classroom observations of the children and their teachers were collected across the length of the study. Teachers were instructed to record themselves and their one or two target students during activities of their choice. In addition, a battery of standardized measures and teacher questionnaires were collected across the length of the study.

The current study included 112 preschool through third grade children with autism (*M*_age_ = 6.18; *SD* = 2.04) and their 57 teachers across 16 districts in **BLIND**. The sample consisted of children from Year 1 of the longitudinal study who met autism criteria on the Autism Diagnostic Observation Schedule, Second Edition (ADOS-2; Lord et al. 2012) and had participated within a classroom video observation at the beginning of the school year. We included only baseline data within the study, data collected at the beginning of the school year and prior to the start of the intervention. This study included video observations collected across a range of special and general classroom settings, including mild/moderate (28%), moderate/severe (39%), autism specific (16%), resource (6%), and inclusive (11%) classroom settings. See Table 1 for information on child characteristics.

**Table 1:** *Child Participant Characteristics & Standardized Measures*

	Mean (SD)	
Age (*n* = 112)	6.18 (2.04)	
Standardized Measures		
Social Affect Total (*n* = 46)	13.89 (3.17)	
Total Comp. Score (*n =* 46)	18.67 (4.06)	
Adaptive Behavior (*n* = 98)	68.44 (14.27)	
Expressive Language (*n* = 109)	40.71 (25.48)	
Receptive Language (*n* = 109)	36.50 (23.25)	
Race/Ethnicity (%)	Percentage	
Black/African American	8%	
Asian/Pacific Islander	5%	
White/Caucasian	38%	
Native American	2%	
Mixed Race	14%	
Other	20%	
Missing	13%	

*Note.* Missing data appeared at to be at random. Social Affect and the total composite score are from the ADOS-2. Adaptive behavior was measured using the Teacher Vineland-II. Receptive and expressive language abilities were measured using the DAS-II

## Standardized Measures and Teacher Questionnaires

**Autism Features.** Autism features were measured using the ADOS-2, a semi-structured “gold standard” diagnostic assessment tool for individuals with autism (Lord et al., 2000). The ADOS-2 utilizes multiple activity models to assess autism features, resulting in three measurements: (1) social affect (SA), (2) restrictive, repetitive behavior (RRB), and (3) a total composite score. We examined the associations among SA and the total composite score with children’s rate of spontaneous communication.

**Adaptive Behavior**. The Vineland Adaptive Behavior Scale (Vineland-II; Sparrow et al., 2005) is an objective measurement of adaptive functioning skills. Using a structured caregiver interview format, three domains are assessed during the evaluation consisting of (1) Communication, (2) Daily Living Skills, and (3) Socialization, with strong reliability (split half reliability estimates are from 0.91 to 0.97). An Adaptive Behavior Composite (ABC) score is provided as an overall assessment of adaptive behavior. We examined the association between the ABC score and children’s rate spontaneous communication.

**Receptive and Expressive Language.** The Differential Abilities Scales, Second Edition (DAS-II; Elliott 2007) measures verbal and nonverbal cognitive functioning in children and adolescents (2–17 years), producing scores on a range of learning processes (Saulnier, 2013). The test produces a general composite score (GCA) and several subtests that comprise three cluster scores: (1) verbal reasoning, (2) nonverbal reasoning and (3) spatial ability. We included age equivalent scores from the verbal comprehension and naming subtest of the verbal reasoning cluster, which represent the median ability of children’s receptive and expressive language skills (Saulnier, 2013). In this study, we examined the associations among children’s receptive and expressive language with the rate of their spontaneous communication.

## Observational Measure and Coding Procedures

We coded the classroom video observations with Noldus Observer® Video-Pro Software (XT 14; 2017) using a multiple-pass procedure, meaning that we coded one child at a time per observation (Kline, 2015). Three trained observers first identified classroom instructional context (small group, whole class, one-to-one) and categorized the observations by activity. We followed the procedures for identifying the amount of time children spent within varying activities as outlined by Sparapani and colleagues (2016), coding six possible activities (literacy, mathematics, other academics, arts and crafts, meals and snacks, and recreation and leisure). We also identified transition time between activities.

Next, five trained undergraduate research assistants coded spontaneous communication using a 2-step process that (1) determined the qualification of the initiation and identified the form of the initiation as described in the *Classroom Measure of Active Engagement* (CMAE; Sparapani et al., 2016), and (2) identified the function of the initiation as guided by the literature. We operationalized spontaneous communication as all instances a child spontaneously directed language (words, pictures, gestures, vocalizations, etc.) toward a peer or teacher for a particular function (Sparapani et al., 2016). We extended upon this definition to identify the varying communicative functions that children initiated within the classroom activities. We identified 10 communicative functions in total, drawing from and extending on the literature to capture the range of communication children within our sample exhibited (e.g., Kim et al., 2014). Within our coding, we identified the following communicative functions: (1) protesting, (2) requesting, (3) initiating social routines, (4) commenting, (5) securing attention, (6) advocating, (7) seeking predictability, (8) using repair strategies, (9) seeking information, and (10) giving and other social initiations. Advocating (communicating thoughts, feelings, and desires to others), seeking predictability (seeking information about the sequence or timing of events), and using repair strategies (using language to repair or prevent a communication breakdown) have rarely been identified within previous studies, however, we felt that including them here was important to capture the full range of communication we observed within our classroom observations. See Table 2 for descriptions of each communicative function and examples.

**Table 2:** *Functions of Spontaneous Communication Initiations – Definitions and Examples*

Function of the Initiation		Verbal and Nonverbal Examples from video observations
**Protesting.** The student verbally (vocalizations, words, and or phrases) or nonverbally (actions, gestures, pictures) protests or refuses by saying “no” or “stop” in some manner to an action, object, or event. Students might, for example, initiate a protest when offered materials or asked to perform an action; How they do so may vary in appearance, but the intention of the act is to indicate “no” or “stop.”		“I can’t,” “I won’t,” “I don’t want to,” “No,” “It’s too long,” “Don’t touch that,” “Stop” Pushing something away or throwing an object to refuse or indicate “no” Purposely hitting or throwing an object toward another person to indicate “I don’t want that” or “I don’t want to” Stopping a partner from moving or touching materials during playtime to indicate “don’t touch that”
**Requesting.** Using words, gestures, phrases, or pictures to direct others’ behaviors. This might include asking others (verbally or nonverbally) to perform an action, or request others to get or give them objects or items.		“I want a blue dice,” “Will you do it? “Can I have pencil?” “I want computer,” “Open” Reaching for an object to indicate “give me” Requesting that others perform actions, such as, “turn the page,” “open the window”
**Initiating Social Routines**. Verbally or nonverbally initiates a turn within an interaction or activity or purposefully performs an action to elicit a response. For example, a student might drop something to get a partner’s reaction.		“My turn,” “Me,” I go next,” “Can’t catch me” (runs away to be chased) Raises hand or pointing on oneself to indicate “my turn” or “pick me” Acting silly to get response—taunting or baiting
**Commenting**. Students verbally or nonverbally show, share, or talk about something that they are doing, seeing, or experiencing. Students, for example, might point to or hold up an item for others to see (“look, she’s got big teeth”), or they might share information (“I’m all done,” “This is easy”).		“It’s blue!,” ”Look,” “This one my grandma bought,” I like Michal Jackson, “It’s a Beta Fish” Pointing to something in the book (“look at how high he’s jumping!”) Holds up fish to show the teacher
**Securing Attention**. Verbally or nonverbally performing actions to gain others’ attention. This might include tapping others or calling their name to gain their attention.		Says “hey” or “excuse me” to gain one’s attention Waves at the teacher or touches her hand to get her attention
**Advocating.** Communicating thoughts, feelings, and desires to others. This might include requesting, suggesting, asking permission, or negotiating a situation in order to get a desired outcome. For example, a student might suggest a change in the schedule or ask permission to continue an activity (“Can I please keep going?). Here students know what they want and use language to negotiate a desired outcome.		“I have an idea, why don’t we eat first and then take the test.” “I’m thirsty, can I go get some water” “Wait, I need to finish reading” “Can we eat snack first and go to writing?” “I feel tired, can I take a break”
**Seeking Predictability**. Seeking information about the sequence or timing of events. Students ask for information related to the daily, lesson, or activity schedule. Students, for example, might ask about why something is different, when something will end, or what will happen next.		“How many more? Are we almost done?” “First this and then we are all done, right?” “What’s next?” Looks at teacher and points to the schedule to inquire about what will happen next.
**Using Repair Strategies**. Using language to repair or prevent a communication breakdown. This may include correcting something that was said wrong or asking for clarification.		“Wait, what about me? (turn was skipped) “No, it’s 300” (correcting teacher) “But, we’re done” (after teacher asks student to solve problem in another way)
**Seeking Information.** Asking questions to achieve more information about a given topic or subject. This might include asking simple (yes/no, “wh”), open-ended, or follow-up questions related to the activity or event that is occurring.		“How come it’s yellow? “What are they eating? (looking at the text)” “Is that a wedding ring box?” “Can those games be on the computer?”
**Giving and other social initiations.** Voluntarily initiates for the purpose of being social with another person, such as giving an object to or performing an action for another person. Students might also a compliment or share an expression with another person after experiencing something together, such as laughing with one another after seeing something funny.		“Here, you can go first” (gives peer the dice during a game), Gives a block to a peer “I’ll help you” (offering help to a friend) Uses language to be polite, such as saying “sorry” to apologize for a behavior or action

## Analytic Methods

**Describing Spontaneous Communication**. We systematically sampled 12-minutes of each classroom video observation for analyses, aiming to capture a range of academic and nonacademic activities across the length of the observation. Fourteen percent (14%) of the observations fell short of the 12-minutes (5:55–11:22). The spontaneous communication codes derived a frequency count of the number of instances that children directed communication (at peers or teachers) for varying functions within the 12-minute sample. We also calculated rate of communication per minute since some videos did not include 12-minutes, as it is a more generalizable metric.

**Child Characteristics.** We provided means and standard deviations of children’s receptive and expressive language, adaptive behavior, and autism features to help characterize the sample. Pearson product-moment correlations were then used to explore the associations among child developmental characteristics and rate of communication.

**Specifying Latent Models Based on Communicative Functions**. We first evaluated the measurement model of spontaneous communication functions as a unidimensional construct, with 10 observed indicators loading onto one single latent factor, as this model represents that most parsimonious latent structure. We then referred to and extended on the literature for guidance on the specification of three competing latent models. We specified a 2-factor model, conceptualizing the 10 communicative functions into two levels of communication sophistication as suggested by Hauck and colleagues (1995). The two latent factors included, *Less Sophisticated* (protesting, requesting, initiating social routines, seeking predictability, and securing attention) and *More Sophisticated* (advocating, using repair strategies, seeking information, commenting, and giving and other social behaviors). Within the 3-factor model, the 10 communicative functions contributed to three latent factors: *“Regulating Others’ Behavior”* (protesting, requesting), *“Showing, Giving, or Drawing Attention toward Oneself”* (initiating social routines, securing attention, commenting, giving and other social behaviors), and *Using Language to Advocate, Repair, or Inquire”* (advocating, seeking predictability, using repair strategies, seeking information). Finally, in the 4-factor model, we modeled “giving and other social behaviors” as a single indicator factor to capture the uniqueness of the intention relative to the others. Hence, the four latent factors included the following dimensions: “*Regulating Others’ Behavior*,” *“Showing or Drawing Attention toward Oneself,”* “*Using Language to Advocate, Repair, or Inquire*,” and *“Giving and Other Social Initiations.”*

## Results

### Interrater Agreement Information – Observational Measures

Observers first achieved interrater rater agreement for activity by coding 10 consecutive video observations at 80% or higher agreement. Once meeting this criteria, interrater agreement among the observers was calculated on 10% of the data and yielded an average percent agreement score of 87% (range 75–95%) and kappa coefficient of 0.85. We then grouped the activities into two primary categories for data analysis; academic activities (literacy, mathematics, and other academics) and nonacademic activities (arts and crafts, meals and snacks, and recreation and leisure).

Interrater agreement for the spontaneous communication variables was achieved using the same criterion outlined above; observers coded 10 consecutive video observations at 80% or higher agreement. Kappa coefficients were not calculated because the data derived frequency counts. We calculated total percentage of agreement (occurrence + nonoccurrence agreements / total Agreements + disagreements) between observers on 15% of these data. Interrater agreement between observers was good overall, yielding an average score of 86% across the spontaneous communication functions (76–100%). This score reflects an average point-by-point agreement between coders at the level of the communicative function, documenting how often observers agreed on occurrences and nonoccurrences of initiations per observation (Yoder et al., 2018). Additional information on the coding procedures can be found in the supplemental materials.

### Descriptive Statistics

**Classroom Activities and Learning Context**. Variability was found in the percentage of classrooms with academic, nonacademic activities, and a mix of academic and nonacademic activities in the sample and by classroom type. Sixteen percent (16%) of our 12-minute sampled observations included both academic and nonacademic activities, although academic activities (literacy, mathematics, other academics) appeared to be most prevalent overall (78%). Classroom context also varied across the classroom video observations, with 54% of the sampled observations including small group contexts, 12% one-to-one, and 10% whole class contexts; 24% of the sampled observations included multiple instructional contexts (e.g., one-to-one and small group). See the supplemental materials for additional information.

**Direction and Function of Spontaneous Communication**. During the 12-minute sample, we found that children initiated 7.53 instances (*SD* = 9.42) of spontaneous communication on average, a rate of 0.69 initiations per minute. This rate, however, varied across the sample, with five children initiating quite often (3.0–5.67 initiations per minute), while 17% of the sample not at all. See the supplemental materials for additional, post hoc analyses. Children most often directed spontaneous communication toward their teachers (83%); 23% of the children directed communication at both their teachers and peers. Two children within the sample only directed communication toward a peer. Eighty-six (86%) of spontaneous communication directed at a peer occurred during small group instruction (14% occurred during whole class instruction).

We observed vast variability in the types and frequency of communicative functions children initiated within activities overall, yet many of the communicative functions were infrequently observed. Commenting was the most frequently observed communicative function relative to all others, (*M* = 3.50; *SD* = 4.86), followed by requesting (*M* = 0.81; *SD* = 1.25), with 58% of the children commenting and 50% requesting at least once during the observation. Initiating social routines, advocating, seeking predictability, and securing attention were rarely observed (fewer than 20% of the sample). See Table 3.

**Table 3:** *Summary Statistics for Initiating Communication Functions*

Communicative Functions	Mean	St dev	Max ^a^	Observed (%)
1. Protesting	0.75	2.11	14.0	26
2. Requesting	0.81	1.25	06.0	50
3. Initiating Social Routines	0.43	1.11	07.0	18
4. Advocating	0.22	0.72	05.0	13
5. Seeking Predictability	0.26	0.69	04.0	15
6. Commenting	3.50	4.86	20.0	58
7. Using Repair Strategies	0.36	0.86	04.0	21
8. Seeking Information	0.37	0.94	05.0	20
9. Securing Attention	0.29	1.01	07.0	14
10. Giving and Other Social Behavior	0.34	0.82	05.0	21

*Note. N* = 112. All data represents frequency counts. Minimum for all variables is 0. Students initiated a total of 7.63 (*SD* = 9.70) initiations on average during the 12-minute sample. “Observed” refers to the percentage of cases that the specific function was observed within the sampled time (1 or more instances). For example, 26% of the children initiated to protest at least 1 time within the sample, 50% initiated to request, 18% initiated a social routine, etc.

**Associations between Child Features and Communication Rate.** We observed significant, negative correlations among ADOS SA (*r* = -0.39, *p* < 0.01) and the total composite score (*r* = -0.31, *p* < 0.05) with rate of communication, indicating a link between fewer autism features and higher rates of communication. We observed significant, positive correlations among receptive (*r* = 0.34, *p* < 0.001) and expressive language (*r* = 0.38, *p* < 0.001) with rate of communication. Finally, we documented a positive, significant correlation between adaptive behavior and communication rate (*r* = 0.30, *p* < 0.01). Small to moderate, significant correlations were documented among children’s varying developmental skills (receptive and expressive language, adaptive function, presence of autism features). See Table 4.

**Table 4:** *Pearson Correlations Among Child Developmental Characteristics and Rate of Communication*

Variable	1	2	3	4	5	6
1. Rate of Communication	—					
2. ADOS Social Affect	− 0.39**	—				
3. ADOS Overall Total	− 0.31*	0.83**	—			
4. Receptive Language	0.34**	− 0.36*	0.42**	—		
5. Expressive Language	0.38**	− 0.33*	− 0.27	0.80**	—	
6. Adaptive Behavior	0.30**	− 0.36*	0.36*	0.48**	0.62**	—

Note.^*^p < 0.05. ^**^p < 0.01

**Fig. 1:**
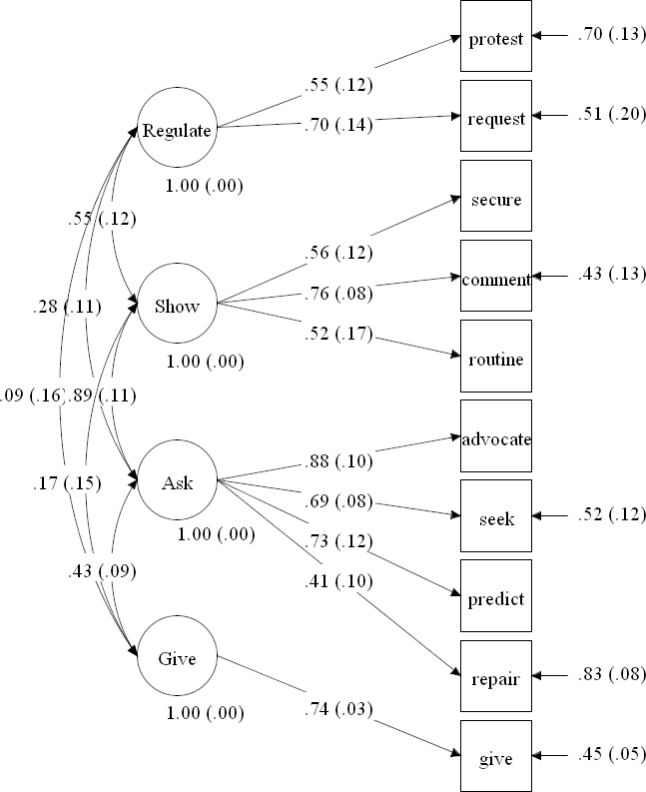
Confirmatory Factor Analysis of Spontaneous Communication Initiations Functions. Regulate (Regulating Others’ Behavior); Show (Showing or Drawing Attention to Oneself); Ask (Using Language to Advocate, Repair, or Inquire); Give (Giving and Other Social Behaviors); Protest (Protesting); Request (Requesting); Secure (Securing Attention); Comment (Commenting); Routine (Initiating Social Routines); Advocate (Advocating); Seek (Seeking Information);Predict (Seeking Predictability); Repair (Using Repair Strategies); Give (Giving and Other Social Behaviors)

**Latent Modeling – Communicative Functions.** Prior to running the factor analyses, we dichotomized initiating social routines, advocating, seeking predictability, and securing attention to prevent model fitting errors because these functions were rarely observed (McCallum et al., 2002). See Table 3. The 4-factor model evidenced good fit to the data, (RMSEA = 0.04 [0.00–0.09]; CFI = 0.96; χ^2^/*df =* 1.19) and the best relative fit compared to the competing models (*p* < 0.05). See Table 5. Each of the 10 factor loadings were significantly different from zero (*p* < 0.01). We observed a strong positive association between the *Showing or Drawing Attention to Oneself* (“Showing”) and the *Using Language to Advocate, Repair, or Inquire (“Asking”)* latent factors (*r* = 0.89). Small to moderate positive associations were observed among the other latent factors (*r* = 0.28–0.55), excluding the associations among the *Giving and Other Social Behaviors* (“Giving”) latent factor with *Regulating Others’ Behavior (“Regulating”) and Showing* (*r* = 0.09, 0.17; *p* > 0.05). See Fig. 1 for detail on the measurement model.

**Table 5:** *Model Fit Statistics using WLSMV Estimation and Difference Testing*

Model Fit Indices		4-Factor		3-Factor	2-Factor		1-Factor	
χ^2^/*df*		1.19		0.78	1.31		0.71	
RMSEA		0.04		0.05	0.05		0.06	
C.I. *P*close-fit H_0_		0.0–0.09 0.57		0.0–0.09 0.47	0.0–0.09 0.43		0.0–0.10 0.31	
CFI		0.96		0.93	0.92		0.90	
Δ χ^2^	4-Factor and 3-Factor 05.88 (*df* = 2), *p* = 0.05		4-Factor and 2-Factor 09.61 (*df* = 4), *p* < 0.05			4-Factor and 1-Factor 14.20 (*df* = 5), *p* = 0.01		

*Note.* Weighted least squares-mean and variance adjusted (WLSMV), Comparative Fit Index (CFI), Root Mean Square Error of Approximation (RMSEA), 90% Confidence Interval, Probability RMSEA < = 0.05 (Pclose-fit H_0_). Model comparison using difference testing against the 4-factor model with the DIFFTEST option in Mplus (Δ χ^2^). The models met the recommended identification assumptions; the model degrees of freedom (*df*) was greater than zero and scaling constraints were imposed on the variances of the latent factors and loadings of the error terms. The four-factor model was identified by fixing the error term of the single indicator factor to equal 1- *r* (S2), where *r* equals reliability (Kline, 2016)

## Discussion

This study provides a detailed examination of how, why, and with whom early school-aged children autism, with a range of verbal abilities, initiate communication within classrooms. Building on past works (e.g., Chiang, 2009; Clifford et al., 2010), we utilized classroom video observations to examine children’s spontaneous communication during academic and nonacademic activities across a range of classroom settings. We explored the relationships among child developmental characteristics, including receptive and expressive language, adaptive behavior, and presence of autism features with the rate of their spontaneous communication initiations—providing initial construct validity to our spontaneous communication variable. Moreover, using confirmatory factor analysis, we investigated the latent structure of spontaneous communication functions within classroom environments. Our study findings contribute to the extant literature by (1) describing, in detail, the spontaneous communication that learners with autism initiate with their teachers and peers across a range of classroom settings and activities, (2) highlighting relationships between children’s developmental characteristics and spontaneous communication, and (3) providing evidence for conceptualizing and measuring spontaneous communication by function in preschool and early elementary-school settings.

### Describing Spontaneous Communication within Classrooms

**Rate of Communication**. Like earlier studies (e.g., Loveland et al., 1988; Stone & Caro-Martinez, 1990), our findings suggest that on average children showed a low rate (0.69 per minute) of spontaneous communication overall within the 12-minute sample, with 17% of the sample not initiating any communication. We found that this was, in part, linked to children’s developmental characteristics—specifically their receptive and expressive language skills, adaptive functioning, and presence of autism features. This low rate of communication we observed in our sample, especially among children who exhibited more need, suggests a need to evaluate how and whether current methods for supporting communication across classroom settings are effective. How are informal and formal communication systems, for example, being utilized during activities to help children initiate communication? Future studies that examine interactions between children and their teachers, rather than solely focusing on child contributions, are also needed in order to determine whether teachers’ talk is an important intervention target, especially in supporting spontaneous communication in learners who communicate less often (Sparapani et al., under review; Sparapani et al., 2020).

Children within our sample most often directed communication toward their teachers, yet we found that 23% directed at least one initiation toward a peer. This is promising, as it appears to be higher than has previously been documented across studies (e.g., Koegel et al., 2012; Taylor et al., 2005). Although future work is needed to disentangle how varying contextual features along with teachers’ talk support and promote peer interactions, our findings highlight small group contexts as a potential contributing factor within classroom activities. This is no surprise, as studies have documented many benefits from small group learning contexts, including opportunities for social interaction among children and high quality teacher-student interactions (e.g., Foorman & Torgesen 2001; Wilson et al., 2012). Studies are needed to outline how learner variability contributes to peer interaction among children with autism, for example, are specific child-level characteristics associated with being involved in peer interactions?

### Measuring Spontaneous Communication Function

Our findings provide initial evidence for conceptualizing a measurement model of spontaneous communication by communicative function in learners with autism within classrooms—proposing a common metric for future studies. We examined the structure of four competing models, drawing from and extending on the current literature. We identified four related yet distinct latent factors; *Regulating Others’ Behavior (Regulating), Showing or Drawing Attention to Oneself (Showing), Using Language to Advocate, Repair, or Inquire (“Asking”), and Giving and Other Social Behaviors (“Giving”).*

These findings provide the groundwork for future studies. We outline a few notable observations regarding the model and highlight areas for future research. The *Giving* latent factor was associated with *Asking*, yet it was not linked with the other latent factors, possibly indicating that children who initiated for *Giving* and *Asking* functions may share commonalities. Further examining the reasons associated with such functions may be important, as studies have suggested that children who ask questions, and interact with their peers, show greater learning gains within classroom lessons (e.g., Connor et al., 2020). Additionally, we observed a high frequency of zeros across the range of functions, indicating a limited range of communicative functions overall. Although we found that child characteristics were linked with communicative function, it is also important to explore interactions between teachers and their students to better understand the learning opportunities that teachers are providing to their students with autism. That is, how are teachers scaffolding interactions with their students with autism to facilitate different communicative functions, such as asking questions or making comments? Finally, understanding whether and how children’s classroom communication, including interactions with peers, is related to their developmental and academic outcomes is an area for future research. If initiating communication more often for a range of functions with both teachers and peers is important for active participation and classroom learning, targeting children’s communication and providing opportunities to communicate for varying functions might be an important area of professional development for classroom teachers.

### Strengths and Limitations

This study has a few limitations. Although we included a large sample of children with autism relative to other studies of its kind, the sample size to parameter ratio is small. Hence, some caution is warranted when interpreting the findings. Future studies should include a larger sample of children, particularly those participating within general education settings since the proportion of special education classroom settings within our study was quite large. Additionally, we sampled 12-minutes of the classroom video observations to capture a range of academic and nonacademic activities across the length of the observation. This sampled time, however, might not reflect the entire duration of the observation. Hence, future work is needed to systematically evaluate and identify sampling procedures that are most reflective of children’s classroom experiences. Regarding age, our study included a narrow age range of children during the early to middle childhood years, yet future research is needed to truly capture middle childhood more broadly (beyond third grade).

Nevertheless, this study had a number of notable strengths. A primary strength included the use of systematic observational methods and sampling procedures to analyze video observations at the child level and capture detail and nuances of child spontaneous communication within a range of classroom activities. A large and heterogeneous sample of children with autism, with a range of autism features, adaptive behaviors, and receptive and expressive language abilities contributes to the overall generalizability of the study findings. Whereas earlier studies evaluating communication in classrooms have emphasized nonacademic activities, such as leisure, free play, and meals in predominantly special education settings (e.g., Dykstra & Watson, 2015), our study included observations of academic activities across a range of classroom settings. Other strengths include the use of reliable and valid measures to help characterize the sample, including the “gold standard” diagnostic measure to confirm autism diagnosis. Interrater agreement between observers was calculated using point-by-point agreement calculations for each of the communicative functions. Finally, the use of latent modeling to examine the factor structure of spontaneous communication for early elementary learners with autism within classrooms is a contribution to the field as it provides initial evidence of validity for conceptualizing the construct as four unique yet related dimensions.

### Educational Implications and Future Directions

Our findings provide initial evidence for a means to conceptualize spontaneous communication by function in learners with autism across varying instructional contexts. Measurement that focuses on function rather than, or in combination with, rate of communication might afford teachers (and researchers) the necessary information for supporting communication development and active participation in learners with autism during classroom activities, as it may be tapping into quality of spontaneous communication initiations. A student, for example, might exhibit a high frequency communication, but looking more closely, the function of these initiations may be limited to behavior regulation. Measuring communicative function might be more beneficial, as it could streamline the measurement process within classrooms and provide teachers with a roadmap outlining their students’ communicative patterns and areas needing targeted support and practice. Since these data might be specific to learners with autism within classroom environments. Future research is needed to replicate these findings using a larger sample of children with autism and including a comparative sample of children without autism across varying instructional contexts. Examining whether potential differences in the measurement model exist by sex (male/female) and classroom setting (general/special education) is another important future direction.

Finally, examining the frequency of children’s spontaneous communication within elementary classrooms may provide a means for measuring children’s joint engagement within classroom activities, which requires one to coordinate attention between people and the environment (Dykstra & Watson, 2015; Sparapani et al., 2016). It is possible, for example, that teachers’ may perceive children’s limited or off-topic spontaneous communication as “disengagement” or problematic behavior, as joint engagement within classrooms might be gauged by paying attention to the task at hand or whatever the teacher is directing child’s attention to. Teachers may perceive such behavior as a form of distractibility, when in reality learners with autism may need additional support to effectively coordinate their attention among people, relevant materials, and/or topics. Hence, understanding and measuring spontaneous communication initiations within classrooms may help educators support children’s joint engagement within activities. Note that, however, measuring other joint attention behaviors in addition to spontaneous communication, such as children’s responses to teachers’ bids, may provide a more accurate representation of joint engagement within classroom activities. This is an area for future research.
